# Autoantibodies to apolipoprotein A-I in hepatitis C virus infection: a role in disease progression?

**DOI:** 10.3389/fimmu.2025.1461041

**Published:** 2025-03-20

**Authors:** Simon H. Bridge, Sabrina Pagano, John K. Lodge, Isaac T. Shawa, Paula Marin-Crespo, Matthew E. Cramp, David A. Sheridan, Simon D. Taylor-Robinson, Nicolas Vuilleumier, R. Dermot G. Neely, Margaret F. Bassendine

**Affiliations:** ^1^ Faculty of Health and Life Sciences, Northumbria University, Newcastle upon Tyne, United Kingdom; ^2^ Translational and Clinical Research Institute, Newcastle University, Newcastle upon Tyne, United Kingdom; ^3^ Division of Laboratory Medicine, Diagnostics Department, Geneva University Hospitals, Geneva, Switzerland; ^4^ Department of Medicine, Medical Faculty, Geneva University, Geneva, Switzerland; ^5^ School of Human Sciences, London Metropolitan University, London, United Kingdom; ^6^ Faculty of Health, Peninsula Medical School, Plymouth University, Plymouth, United Kingdom; ^7^ Department of Biomedical and Forensic Science, University of Derby, Derby, United Kingdom; ^8^ Department of Surgery and Cancer, Faculty of Medicine, Imperial College London, London, United Kingdom; ^9^ Department of Blood Sciences, Newcastle upon Tyne Hospitals NHS Foundations Trust, Newcastle upon Tyne, United Kingdom

**Keywords:** hepatitis C virus (HCV), autoantibodies, apolipoprotein A-I, disease progression, cirrhosis

## Abstract

**Background:**

Chronic HCV (CHC) infection is associated with autoimmunity. IgG autoantibodies to apolipoprotein A-I (AAA-I) predict all-cause mortality. We evaluated AAA-I in CHC patients and in those who were not viraemic, either because of spontaneous resolution (SR) of infection or HCV clearance following sustained virological response (SVR) after interferon therapy. We limited the study to HCV genotypes 1 and 3, the dominant HCV genotypes circulating in the UK.

**Methods:**

Serum samples from 126 CHC patients and 114 nonviraemic individuals (25 SR and 89 SVR) were assayed for AAA-I and lipoproteins. AUC was calculated for AAA-I and HDL-related parameters and used to predict cirrhosis. Fibronectin (FN) and FN-mRNA were measured in human hepatic stellate cells (LX-2) in the presence or absence of AAA-I.

**Results:**

AAA-I was found in 47% of patients with CHC, 37% of SVR patients, and 16% of SR individuals (CHC vs. SR, *p* = 0.004). AAA-I levels in CHC patients were higher in those with cirrhosis (*p* = 0.0003). The AUC for AAA-I, apoA-I, and HDL-C in predicting cirrhosis was 0.72 (*p* < 0.001), 0.65 (*p* = 0.01), and 0.64 (*p* = 0.02). After 48 h in the presence of AAA-I, LX-2 cells showed an 80% increase in FN-mRNA compared to the LX-2/IgG control (*p* = 0.028) and higher levels of FN (*p* = 0.0016).

**Conclusions:**

CHC is often associated with AAA-I, and these can persist after SVR. AAA-I is a robust predictor of cirrhosis in CHC infection. LX-2 cells exposed to AAA-I showed increased FN. Further studies are warranted to define the role of AAA-I in promoting not only viral persistence but also fibrosis.

## Introduction

The coronavirus disease 2019 (COVID-19) pandemic has highlighted the importance of improving our understanding of the association between viral infections and autoimmunity (1). It has long been recognized that the hepatitis C virus (HCV) is associated with the development of new-onset autoimmune disorders, such as thyroiditis (2). Chronic hepatitis C (CHC) infection remains a global challenge, with an estimated 400,000 HCV-related deaths annually (3) and 71 million persons remaining untreated, despite the significant medical advance of highly effective direct-acting antiviral agents (DAAs) (4). In addition, HCV rarely induces sterilizing immunity, and those cured with DAAs remain at risk of reinfection (3). Even among people successfully treated with DAAs, mortality rates remain higher than those in the general population (5). We have previously reported a high prevalence of autoantibodies to apolipoprotein A-I (AAA-I), which predict all-cause mortality in the community (6), in patients with advanced CHC receiving DAAs (7). COVID-19 also induces a marked, though transient, AAA-I response and has been reported to predict persistent respiratory symptoms (8). In this study, we aimed to address whether AAA-I were present in a larger cohort of individuals with HCV infection including those who were previously infected but no longer viraemic, either due to spontaneous resolution (SR) of acute infection or HCV clearance following successful interferon-based antiviral therapy (sustained virological response [SVR]).

## Materials and methods

### Study participants

Sera were obtained from HCV Research UK (HCVRUK) biobank following informed consent and approval from the HCVRUK biobank access committee, as approved by the NRES Committee East Midlands (REC 11/EM/0314) ethical review board. Sera were also obtained from subjects attending clinics at the University Hospitals Plymouth NHS Trust following informed consent and approval by the NRES Committee South West and Plymouth (REC 1703) ethical review board. Serum was used, as it is the matrix of choice for the detection of AAA-I (9). The study cohort comprised three groups: (1) 126 untreated individuals with CHC (61 infected with genotype 1 (GT1) and 65 with genotype 3 (GT3); (2) HCV antibody-positive, HCV RNA-negative individuals with spontaneous resolution (*n* = 25, the SR group); and (3) 89 patients who were HCV RNA negative following a SVR to interferon-based antiviral therapy.

### Lipid profiles

Total cholesterol (TC), triglyceride (TG), and HDL-C were measured by automated enzymatic methods using an Olympus AU 640 Analyzer (Olympus, Watford, UK). Non-HDL-C concentrations were calculated by subtracting the HDL-C from TC, which may be more accurate in estimating lipid-related cardiovascular (CV) risk (10). ApoA-I and apoB were measured by automated immunoturbidometric methods on a Roche Cobas Modular c702 Analyzer (Roche Diagnostics, Lewes, UK).

### AAA-I ELISA

Serum samples were blinded, and AAA-I was determined using a well-validated ELISA, as previously described (7, 11). Briefly, Maxi-Sorp plates (Nunc™, Roskilde, Denmark) were coated with purified, human-derived delipidated apoA-I (20 µg/mL; 50 μL/well) for 1 h at 37°C. After three washes with phosphate-buffered saline (PBS)/2% bovine serum albumin (BSA; 100 μL/well), all wells were blocked for 1 h with 2% BSA at 37°C. Samples were diluted 1:50 in PBS/2% BSA and incubated for 1 h. Additional patient samples at the same dilution were also added to an uncoated well to assess individual nonspecific binding. After six further washes, 50 μL/well of signal antibody (alkaline phosphatase-conjugated antihuman IgG; Sigma-Aldrich, Gillingham, Dorset, UK) diluted 1:1,000 in PBS/2% BSA solution, was incubated for 1 h at 37°C. After six more washes (150 μL/well) with PBS/2% BSA solution, the phosphatase substrate *p*-nitrophenyl phosphate disodium (50 μL/well; Sigma-Aldrich), dissolved in diethanolamine buffer (pH 9.8), was added. Samples were tested in duplicate, and the absorbance was determined at an optical density (OD) of 405 nm (Filtermax 3, Molecular Devices™, San Jose, CA, USA) after 20 min incubation at 37°C. The corresponding nonspecific binding value was subtracted from the mean absorbance value for each sample. To be defined as AAA-I seropositive, samples had to show absorbance values > 0.6 and index values > 37% (11, 12).

### Cell culture

Human hepatic stellate (hHSC) LX-2 cells, purchased from Merck Millipore (Sigma-Aldrich, Burlington, MA, USA; cat. SCC064), were seeded at 7,000 cells/well in Iscove’s modified Dulbecco’s medium (IMDM, supplemented with 20% fetal bovine serum, 1% penicillin–streptomycin, 1% l-glutamine, 1% nonessential amino acids, and 1% sodium pyruvate [Gibco BRL-Life Technologies, Rockville, MD, USA]) in 48-well plates (Corning Inc., Corning, New York, USA). The following day, cells were either incubated with 40 μg/mL of goat polyclonal antihuman apoA-I IgG (11AG2; Academy Bio-Medical Company, Houston, TX, USA) or 40 μg/mL goat control IgG (A66200H; Meridian Life Science, Memphis, TN, USA) in serum-free IMDM for 48–72 h. At the indicated time points, cell supernatants were collected, and RNA was extracted after 48 h using the Total RNA Purification Micro-RNAeasy Kit (Qiagen, Hilden, Germany) according to the manufacturer’s protocol.

### Quantitative PCR

For mRNA quantification, cDNA was synthesized using the Improm-II Reverse Transcription System (Promega, Madison, WI, USA). Quantitative real-time PCR was performed in duplicate with TaqMan Universal Master Mix II (no UNG) on the StepOne Plus System (Thermo Fisher Scientific, Waltham, MA, USA). The mRNA levels were normalized to the housekeeping gene, glyceraldehyde-3-phosphate dehydrogenase (GAPDH, hs99999905_m1). The following primer was used for the detection of fibronectin (HS01549976-m1) (Applied Biosystems, Foster City, CA, USA). Data were analyzed with the 7900HT SDS Software v2.3 (Applied Biosystems). Relative expression of mRNA was calculated using the comparative threshold cycle (CT) method (2−ΔΔCt) (13).

### Fibronectin concentration

Fibronectin was measured in LX-2 supernatants diluted 1:50 (v/v) using a Magnetic Luminex Assay (R&D systems, Minneapolis, MN, USA) according to the manufacturer’s protocol, with a Bio-Plex 200 array reader (Bio-Rad Laboratories, Hercules, CA, USA) and Luminex MAP™ Technology (Luminex Corporation, Austin, TX, USA).

### Statistical analyses

Univariate statistical analysis was performed using Minitab v.18 (Minitab Ltd, Coventry, UK) and Prism 9 (GraphPad, San Diego, CA, USA). The distribution of continuous variables was assessed by Anderson–Darling normality tests. Continuous parametric variables were presented as the mean ± SD, while nonparametric variables were presented as the median and interquartile ranges (IQR). The significance of differences between two parametric groups was performed by using either an unpaired two-sample *t*-test or a Welch’s *t*-test. For two nonparametric group comparisons, a Kruskal–Wallis test or Mann–Whitney *U* test was used. For multiple group comparisons, *p*-values were calculated using either a one-way ANOVA with Sidak’s multiple comparison test for parametric variables or a Kruskal–Wallis test with Dunn’s multiple comparisons test for nonparametric variables. Correlation analysis was performed using either Pearson’s correlation test for parametric data or Spearman rank correlation test. Two-sided Fisher’s exact test or Chi-square test was used to compare categorical variables. The discriminatory power of AAA-I, AAA-I/ApoA-I ratio, and HDL-C-related parameters for predicting cirrhosis were assessed using receiver operating characteristic (ROC) analysis. ROC curves were plotted, and diagnostic parameters such as sensitivity, specificity, area under the curve (AUC), and 95% CI were calculated using the Wilson/Brown method.

## Results

### Study participants

The demographic, clinical, and biochemical characteristics of the 240 study participants are summarized in **Table 1**, **Supplementary Figure S1**. Most patients with CHC were men (67%) and older (> 47 years), with 30 individuals having cirrhosis (24%). They also had lower serum concentrations of TC, TG, and apoB, as well as lower ratios used in CV risk assessment (14), including a lower apoB/apoA-I ratio, TG/HDL-C ratio, and TC/HDL-C ratio compared to those without viraemia (SR and SVR). The clinical characteristics of chronic HCV patients infected with either GT1 or GT3 were compared to those of the SVR group of previously GT1- or GT3-infected patients following virological cure, as shown in **Table 2**. These differences were more pronounced in patients infected with GT3. In total, 41 participants had cirrhosis (diagnosed by liver histology and/or liver imaging; 30 CHC and 11 SVR).

**Table 1:** Clinical characteristics of the study cohort.

Characteristic	SR (n = 25)	CHC (n = 126)	SVR (n = 89)	*p*
Sex, F n (%)	6 (24%)	41 (33%)	19 (21%)	0.195
Age (yr), median (IQR)	34 (8.5)	47 (13.5)	40 (13.5)	**<0.001**
BMI (kg/m^2^), mean ± SD	25.54 ± 4.23	24.13 ± 3.13	26.22 ± 4.28	**<0.001**
Ethnicity n (%) Asian - Chinese Asian - Indian Asian - Pakistani White - Other White - British White - Irish Other ethnic group Not known	0 (0%) 0 (0%) 1 (4%) 1 (4%) 14 (56%) 0 (0%) 0 (0%) 9 (36%)	1 (0.79%) 2 (1.59%) 7 (5.55%) 9 (7.14%) 106 (84.13%) 1 (0.79%) 0 (0%) 0 (0%)	0 (0%) 2 (2.25%) 9 (10.11%) 13 (14.61%) 63 (70.79%) 1 (1.12%) 1 (1.12%) 0 (0%)	ND
Smoking status; n (%) Current Past/Former Never Not available	13 (52%) 1 (4%) 2 (8%) 9 (36%)	88 (70%) 25 (20%)13 (10%) 0 (0%)	59 (66%) 16 (18%)12 (13%) 2 (2%)	ND
HCV GT 1/3, n	N/A	61/65	46/43 (formerly infected)	ND
HCV RNA (_log10_ IU/mL)	N/A	6.00 ± 0.75	N/A	ND
HIV-1 co-infection; n (%)	1 (4%)	2 (1.6%)	2 (2.25%)	0.519
Cirrhosis; n (%)	0 (0%)	30 (23.81%)	11 (12.36%)	**0.002**
Liver transplant; n (%)	0 (0%)	1 (0.8%)	2 (2.25%)	0.691
Diabetes; n (%)	0 (0%)	10 (7.94%)	4 (4.5%)	0.311
Cryoglobulinaemia; n (%)	0 (0%)	2 (1.6%)	1 (1.1%)	0.999
Total cholesterol (mmol/L)	4.40 (1.55)	3.9 (1.10)	4.4 (1.20)	**0.0002**
Non-HDL-C (mmol/L)	3.35 (1.53)	2.75 (1.20)	3.30 (1.30)	**<0.0001**
Triglyceride (mmol/L)	1.75 (1.08)	1.00 (0.50)	1.5 (0.85)	**<0.0001**
HDL-C (mmol/L)	1.00 (0.30)	1.10 (0.50)	1.00 (0.30)	**0.032**
Apolipoprotein B (g/L)	0.81 (0.39)	0.76 (0.29)	0.89 (0.32)	**<0.0001**
Apolipoprotein A-I (g/L)	1.32 (0.24)	1.47 (0.50)	1.37 (0.40)	0.148
ApoB/A-I ratio	0.60 (0.41)	0.51 (0.26)	0.66 (0.32)	**<0.0001**
TG/HDL-C ratio	1.62 (1.92)	0.91 (0.96)	1.32 (1.10)	**<0.0001**
TC/HDL-C ratio	4.78 (2.40)	3.46 (1.67)	4.27 (2.04)	**<0.0001**

BMI, body mass index; CHC, chronic hepatitis C; IQR, interquartile range; ND, not determined; SR, spontaneous resolver; SVR, sustained virological response.

Parametric variables are presented as mean ± standard deviation. Nonparametric variables are presented as median (interquartile range). One-way analysis of variance (ANOVA) with Sidak’s multiple comparisons test was used to compare three mean values per group for parametric variables. Kruskal–Wallis ANOVA test with Dunn’s multiple comparisons test was used to compare three median values for nonparametric variables; 3 × 2 contingency table Fisher’s exact test was used to compare categorical groups. For the multiple comparison tests of continuous variables, see **Supplementary Figure S1**. *p* < 0.05 were considered statistically significant and shown in bold font.

**Table 2:** Clinical characteristics of the cohort with chronic HCV genotype 1 or genotype 3 infection compared to sustained virological responders.

Characteristic	HCV genotype 1		*p*-value	HCV genotype 3		*p*-value
	CHC (*n* = 61)	SVR (*n* = 46)		CHC (*n* = 65)	SVR (*n* = 43)	
Sex: F (*n* (%))	20 (33)	8 (17)	0.081	21 (32)	11 (26)	0.522
Age (year)	47 (15.0)	42 (14.3)	**0.011**	47 (13.0)	38 (12.0)	**< 0.001**
BMI (kg/m^2^)	24.1 ± 3.0	25.4 ± 4.7	0.103	24.2 ± 3.3	27.0 ± 3.65	**< 0.001**
Total cholesterol (mmol/L)	4.20 (1.20)	4.60 (1.20)	0.085	3.70 (1.00)	4.35 (1.23)	**< 0.001**
Non-HDL-C (mmol/L)	3.00 (1.15)	3.60 (1.40)	**0.023**	2.60 (1.10)	3.10 (1.28)	**< 0.001**
Triglyceride (mmol/L)	1.10 (0.80)	1.40 (0.90)	0.147	1.00 (0.50)	1.50 (0.80)	**< 0.001**
HDL-C (mmol/L)	1.20 (0.65)	1.00 (0.40)	0.068	1.10 (0.40)	1.00 (0.30)	0.293
ApoA-I (g/L)	1.55 (0.46)	1.39 (0.43)	0.089	1.40 (0.38)	1.37 (0.40)	0.995
ApoB (g/L)	0.80 ± 0.24	0.92 ± 0.24	**0.013**	0.74 ± 0.23	0.92 ± 0.24	**< 0.001**
ApoB/apoA-I	0.49 (0.24)	0.64 (0.27)	**0.004**	0.52 (0.29)	0.66 (0.32)	**0.004**
TC/HDL-C	3.46 (1.73)	4.29 (2.08)	**0.005**	3.46 (1.64)	4.18 (1.97)	**0.002**
TG/HDL-C	1.00 (1.07)	1.16 (1.27)	0.078	0.91 (0.81)	1.36 (1.09)	**0.001**
AAA-I+ (*n* (%))	28 (45.90)	18 (39.13)	0.556	31 (47.7)	18 (39.13)	0.562
AAA-I (%), median (IQR)	32.0 (29.6)	35.6 (22.3)	0.580	36.26 (20.8)	30.8 (23.5)	0.066

BMI, body mass index; CHC, chronic hepatitis C; IQR, interquartile range; SR, spontaneous resolver; SVR, sustained virological response.

Parametric variables are presented as mean ± standard deviation. Nonparametric variables are presented as median (interquartile range). Unpaired *t*-tests were used to calculate *p*-values for comparisons between parametric variables. Kruskal–Wallis tests were used to calculate *p*-values for comparisons between nonparametric variables. Fisher’s exact test was used to compare categorical groups. *p* < 0.05 were considered statistically significant and shown in bold font.

### AAA-I is highest in chronic HCV infection

**Figure 1:**
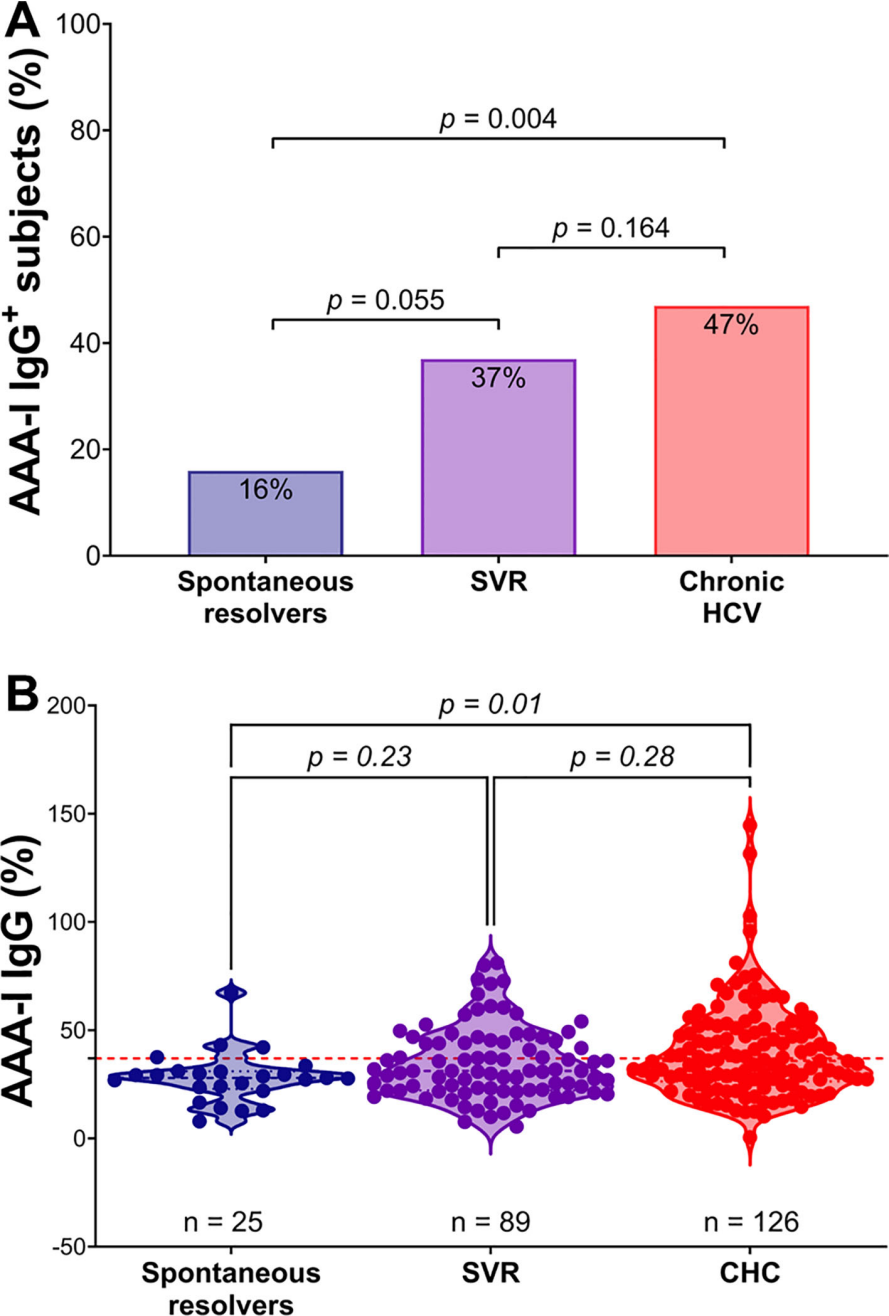
Comparison of AAA-I responses in the study cohort. **(A)** Percentage of AAA-I+ subjects in each group: 4/25 (16%) in the SR group, 33/89 (37%) in the SVR group, and 59/126 (47%) in the CHC group. For proportional comparisons, *p* values were calculated using Fisher’s exact tests. **(B)** Violin plots showing AAA-I levels in SR, CHC, and SVR groups. For statistical differences, *p* values were calculated using a Kruskal–Wallis ANOVA test with Dunn’s correction for multiple comparisons.

In the SR group, four of 25 (16%) were AAA-I positive, which is similar to the 19.9% previously reported in the general population (15). In the CHC group, 59/126 (47%) were AAA-I positive (28/59 for GT1 [46%] and 31/59 for GT3 [48%]), while in the SVR group, 33/89 (37%) were AAA-I positive (**Figure 1A**). There was a significantly higher proportion of AAA-I-positive subjects in the CHC group than in the SR group (*p* = 0.004). There were similar proportions of AAA-I-positive individuals in the CHC and SVR groups (*p* = 0.164), but the difference in the proportion of AAA-I-positive individuals between the SR and SVR groups was not significant (*p* = 0.055). The magnitude of AAA-I responses was compared between groups (**Figure 1B**); AAA-I levels were significantly higher in the CHC compared to the SR (35.8% vs. 28%; *p* = 0.01).

### The impact of AAA-I on virological and lipid parameters

**Figure 2:**
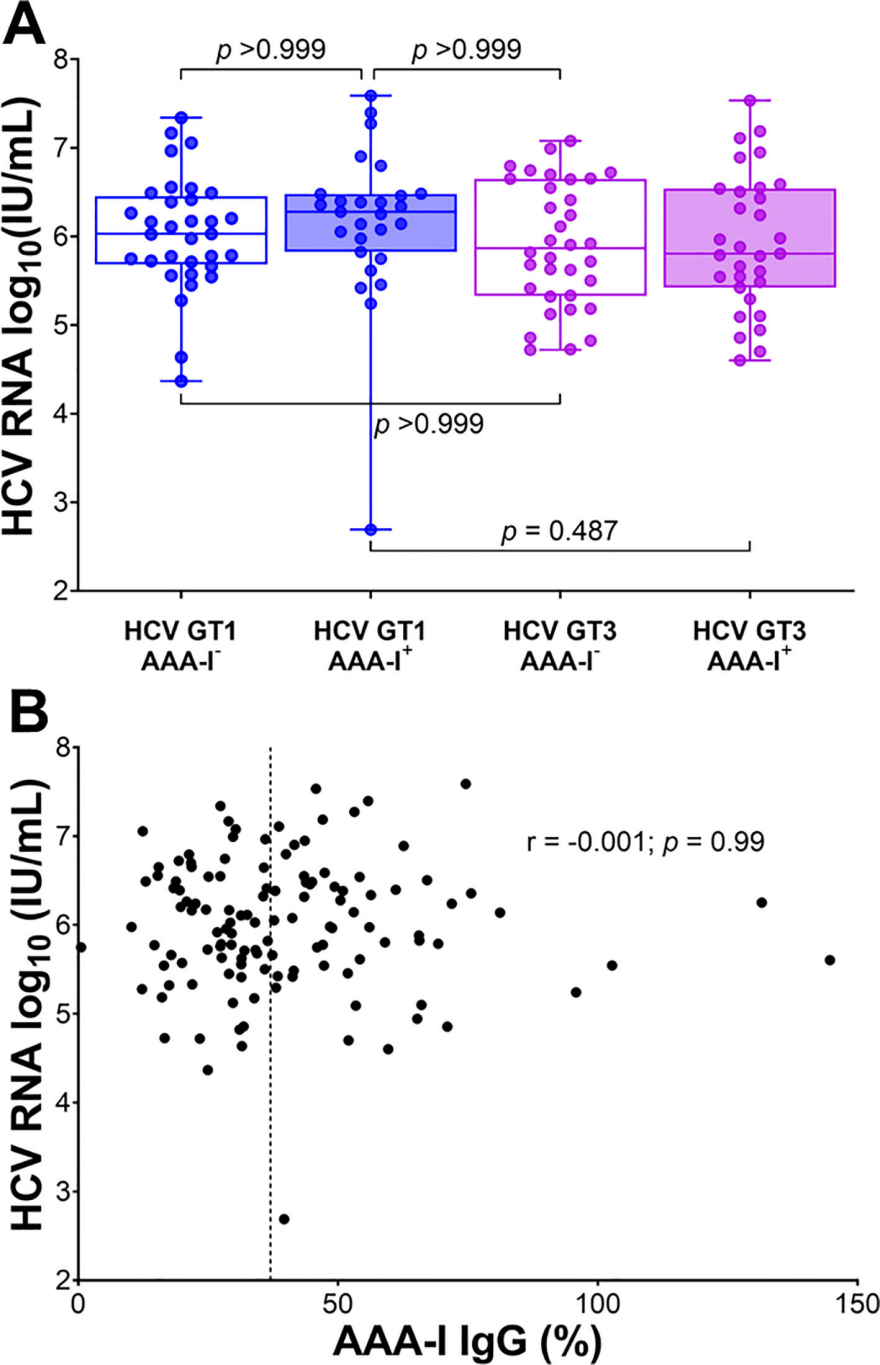
The relationship between AAA-I and HCV RNA viral loads. **(A)** Boxplots showing the impact of AAA-I on HCV viral loads in HCV GT1 and GT3 infections, with significance determined by the Kruskal–Wallis ANOVA test with Dunn’s correction for multiple comparisons. **(B)** Scatterplot showing the strength and significance of the association between HCV viral load and AAA-I levels; *r* and *p*-values were calculated using a Spearman rank correlation test. The dashed line at 37% indicates AAA-I positivity.

There were no significant differences in HCV viral loads between AAA-I seropositive and seronegative groups (Welch’s *t*-test, 5.97 vs. 6.04 log_10_ (IU/mL); *p* = 0.627). Viral genotype did not affect this overall result (**Figure 2A**). There was also no correlation between HCV viral loads and the magnitude of AAA-I response (**Figure 2B**; *r* = − 0.001; *p* = 0.99).

We evaluated the association between AAA-I levels and total cholesterol, non-HDL-C, and HDL-related parameters (see **Supplementary Figure S2**). Overall, there was a significant inverse relationship between the magnitude of AAA-I levels and total cholesterol (*r* = − 0.142; *p* = 0.030), but not with HDL-C (*r* = − 0.033; *p* = 0.616) or apoA-I (*r* = − 0.112; *p* = 0.086). A similar association was also found in the CHC group between AAA-I levels and total cholesterol (*r* = − 0.182; *p* = 0.043), but not with HDL-related parameters.

### AAA-I and cirrhosis

**Figure 3:**
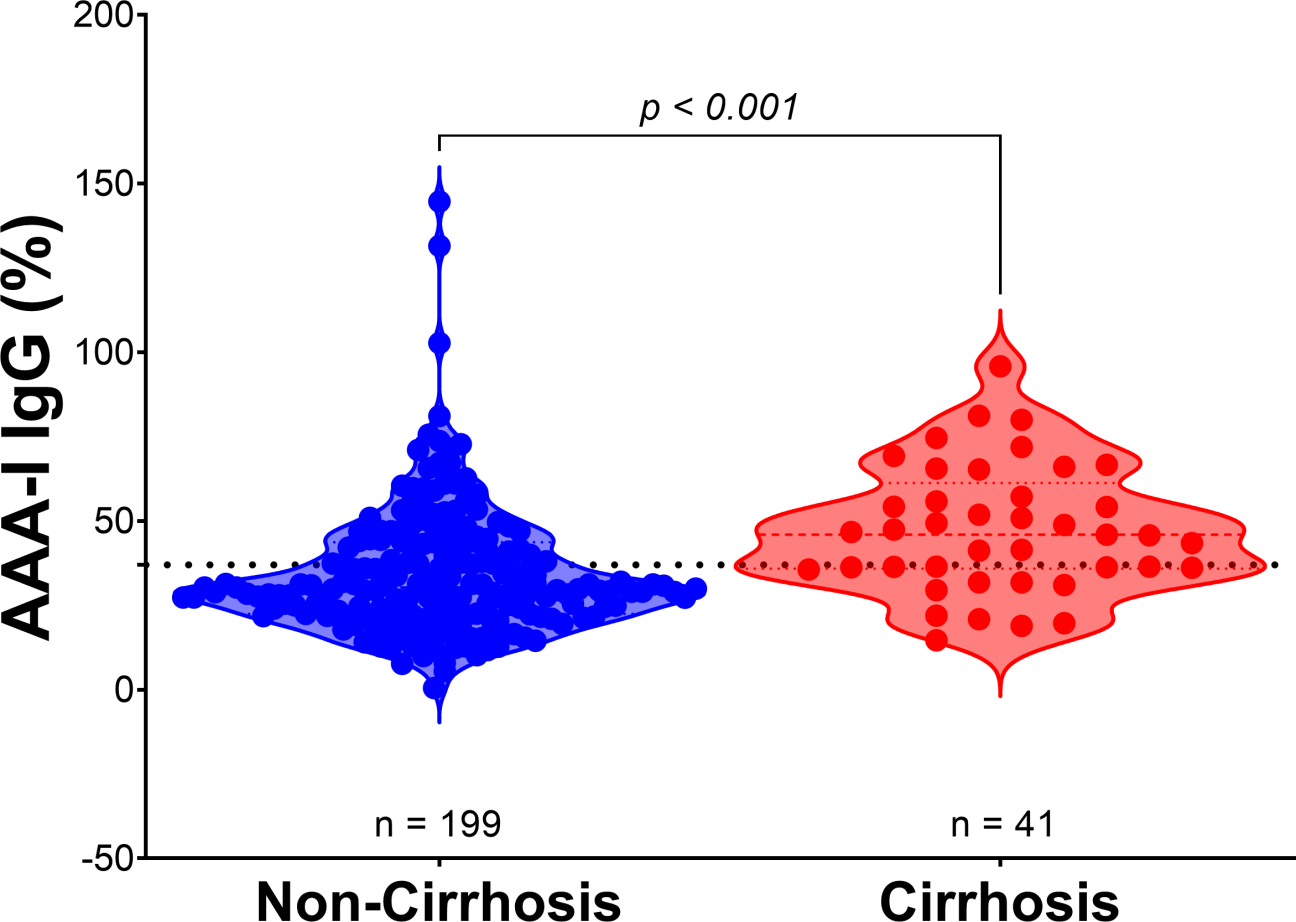
AAA-I levels in patients with cirrhosis compared to those without cirrhosis. Violin plots show the difference between AAA-I levels in individuals without cirrhosis (*n* = 199) and those with cirrhosis (*n* = 41), with proportions of 30.3% vs. 46.0%. Statistical significance was determined using the Mann–Whitney *U* test (*p* < 0.001).

In the total cohort, we found AAA-I levels were significantly higher in patients with cirrhosis (**Figure 3**; 46.0% vs. 30.3%; *p* < 0.001). There was no association between AAA-I levels and the individual’s age at sampling (*r* = 0.081; *p* = 0.213). To further investigate the impact of age, we compared the ages of AAA-I-positive (*n* = 96) to AAA-I-negative (*n* = 144) individuals and found no significant difference (43.6 ± 10.2 year vs. 41.9 ± 9.7 year; *p* = 0.224).

**Figure 4:**
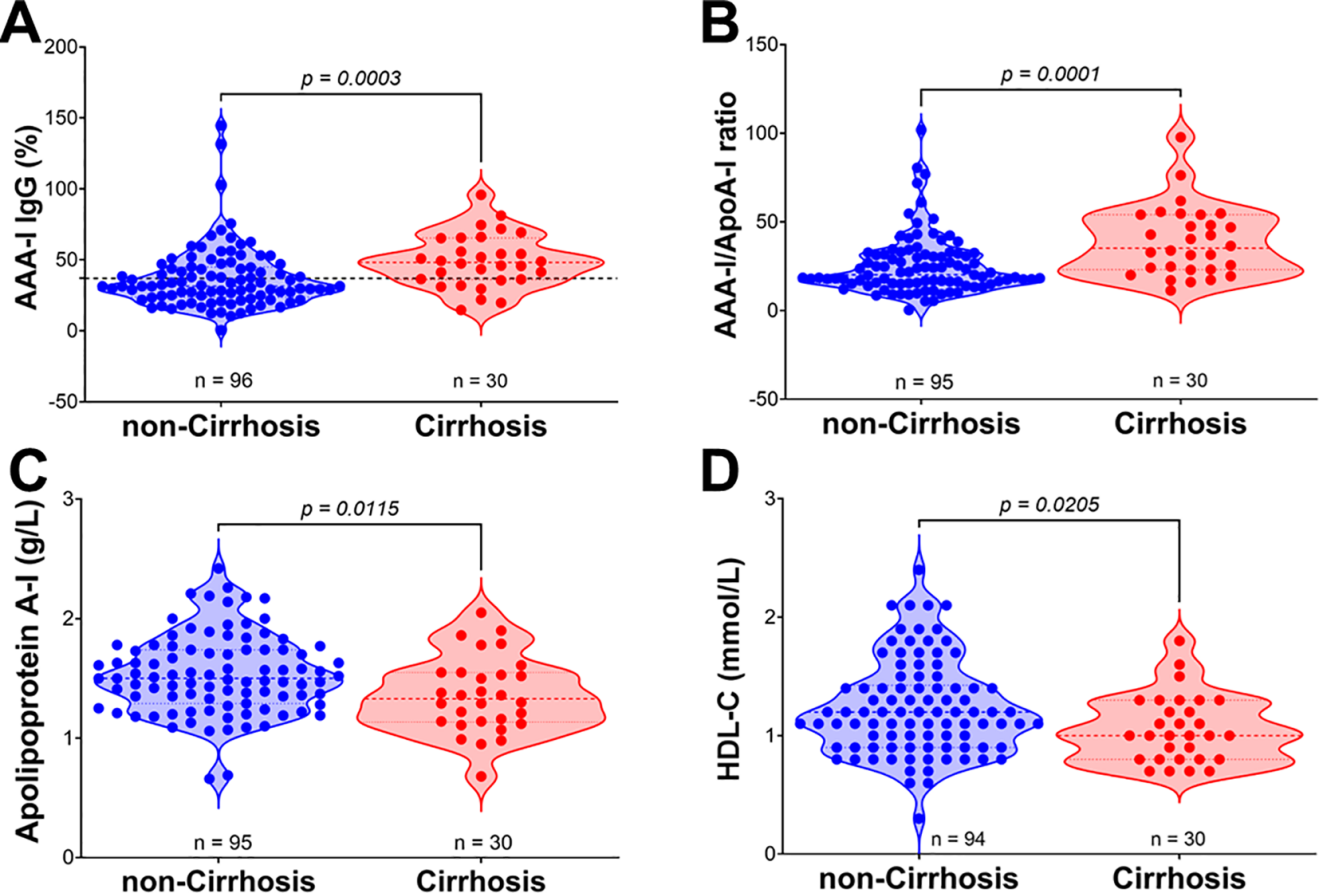
Comparison of AAA-I, AAA-I/ApoA-I ratio, and HDL-related parameters in chronic HCV patients with and without cirrhosis. **(A)** AAA-I (%), 31.46% vs. 48.15%; Mann–Whitney *U* test, *p* = 0.0003. **(B)** AAA-I/ApoA-I ratio, 20.2 vs. 35.2; Mann–Whitney *U* test, *p* = 0.0001. **(C)** Apolipoprotein A-I, 1.53 g/L vs. 1.36 g/L; unpaired *t*-test, *p* = 0.0115. **(D)** HDL-C, 1.20 mmol/L vs. 1.00 mmol/L; Mann–Whitney *U* test, *p* = 0.0205.

In the CHC group (**Figure 4**), we found significant differences in the magnitude of the AAA-I response between patients with or without cirrhosis (48.15% vs. 31.5%; *p* = 0.0003). We also found significant differences in the AAA-I/ApoA-I ratio (35.2 vs. 20.2; *p* = 0.0001). As expected, concentrations of apoA-I (1.36 g/L vs. 1.53 g/L; *p* = 0.0115) and HDL-C (1.06 mmol/L vs. 1.24 mmol/L; *p* = 0.0205) were lower in those with cirrhosis. In the SVR group (**Supplementary Figure S3**), the only significant difference between patients with or without cirrhosis was the AAA-I/ApoA-I ratio (30.4 vs. 22.6; *p* = 0.0322).

**Figure 5:**
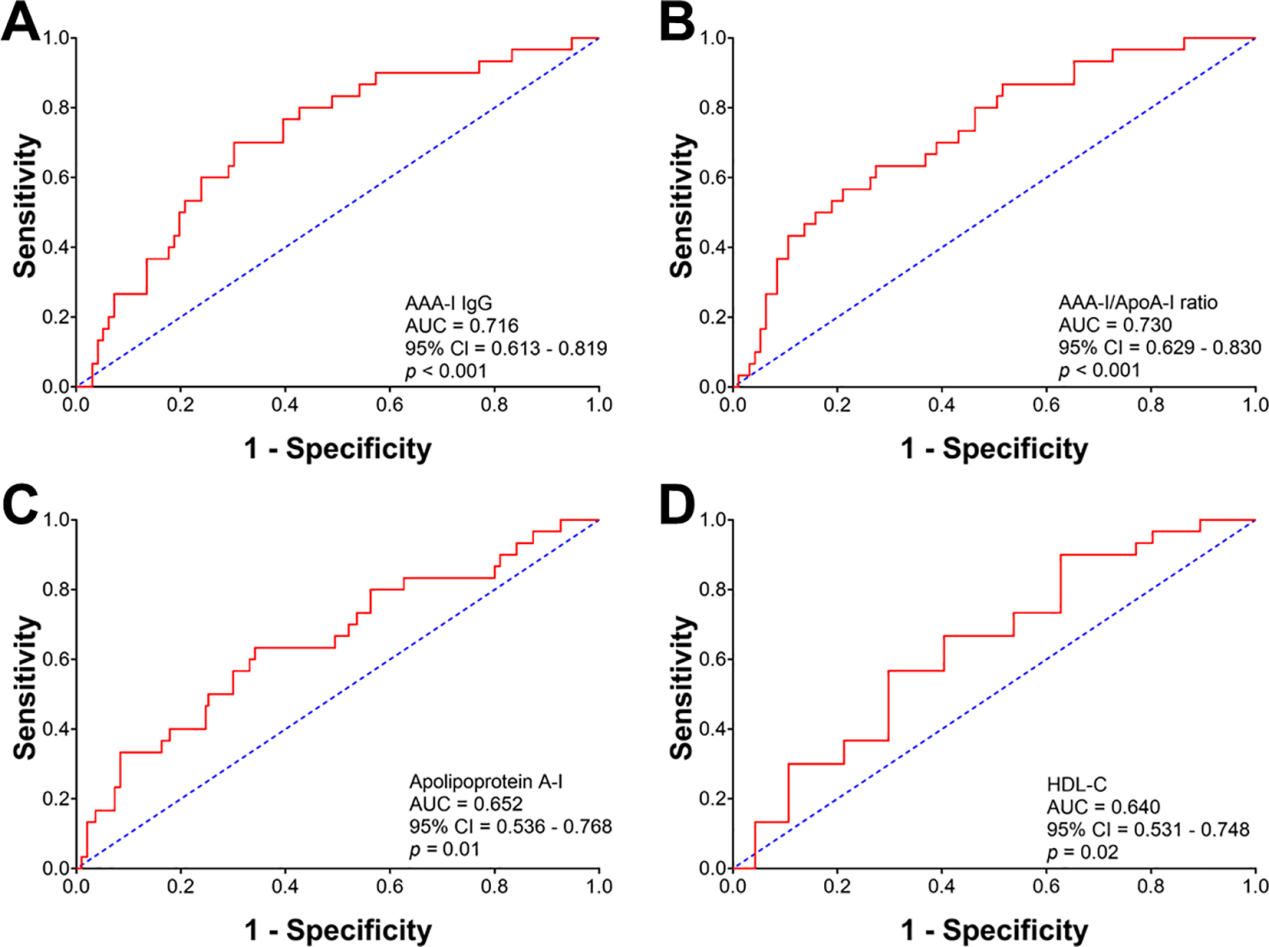
Receiver operating characteristic curve analysis evaluating the predictive robustness of AAA-I, AAA-I/ApoA-I ratio, and HDL-related parameters for cirrhosis in chronic HCV patients. **(A)** AAA-I; AUC: 0.716, *p* < 0.001. **(B)** AAA-I/ApoA-I ratio; AUC: 0.730, *p* < 0.001. **(C)** Apolipoprotein A-I; AUC: 0.652, *p* = 0.01. **(D)** HDL-C; AUC: 0.640, *p* = 0.02.

ROC analyses were performed to determine the diagnostic power of AAA-I, AAA-I/ApoA-I, and HDL-related parameters in predicting cirrhosis in CHC patients (**Figure 5**). The most robust predictors of cirrhosis in CHC patients were AAA-I (AUC: 0.716, *p* < 0.001) and the AAA-I/ApoA-I ratio (AUC: 0.730, *p* < 0.001). HDL-related parameters were also significant predictors of cirrhosis in CHC: apoA-I (AUC: 0.652, *p* = 0.01) and HDL-C (AUC: 0.640, *p* = 0.02). We also performed ROC analyses with the same parameters in the SVR group (**Supplementary Figure S4**) and found that the only parameter predictive of cirrhosis was the AAA-I/ApoA-I ratio (AUC: 0.699; *p* = 0.03).

In a subgroup of 30 patients (28 CHC and two SVR) in whom FibroScan™ scores (Echosens, Paris, France) (16) were available (**Supplementary Figure S5**), we evaluated whether AAA-I was associated with increased liver stiffness measurements using the cut-off value of 12.9 kPa to indicate cirrhosis (17). This cut-off was used as it has been shown to have high diagnostic accuracy for cirrhosis in a large multicenter study of 1,839 patients. AAA-I-positive patients had significantly higher measurements of liver stiffness than those who were AAA-I negative (8.7 kPa vs. 4.9 kPa, *p* = 0.0437).

### AAA-I and HCV clearance with antiviral therapy

**Figure 6:**
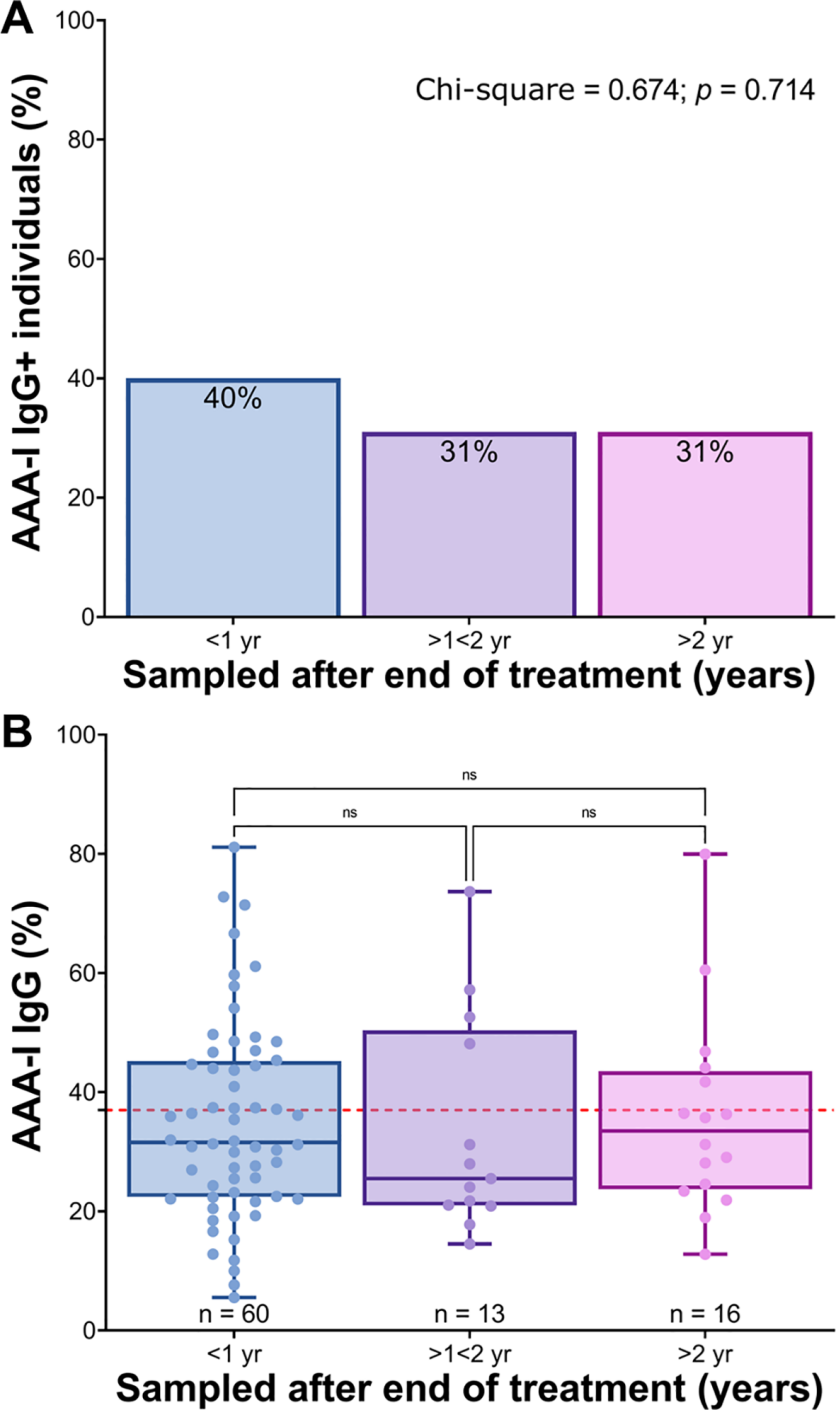
Durability of the AAA-I response following interferon-based treatment. Samples were stratified into three groups after interferon-based treatment: (1) < 1 year after treatment (median sampling time was 0.5 years), (2) > 1 but < 2 years after treatment (median sampling time was 1.1 years after treatment) and (3) > 2 years after treatment (median sampling time was 3.9 years after treatment). **(A)** Comparison of AAA-I seropositive individuals at each sampling time point following IFN therapy cessation. Significance was determined using a Chi-square test; 40% vs. 31% vs. 31%, *p* = 0.714. **(B)** Comparison of AAA-I levels at each sampling time point following antiviral therapy. Significance was assessed using the Kruskal–Wallis ANOVA test with Dunn’s correction for multiple comparisons. No significant differences were observed.

Overall, 33/89 (37%) individuals who were no longer viraemic after SVR had a positive AAA-I (**Figure 6**). The majority of sera were taken ≤ 1 year posttreatment, and in those individuals, 24/60 (40%) were AAA-I positive. AAA-I was detected in four of 13 (31%) samples taken after 1 year but before 2 years posttreatment and five of 16 (31%) samples obtained in > 2 years after cessation of antiviral therapy. No significant differences were found in either AAA-I prevalence (*p* = 0.714) or levels between these four time points postinterferon-based treatment.

### Hepatic stellate cells in the presence of AAA-I

**Figure 7:**
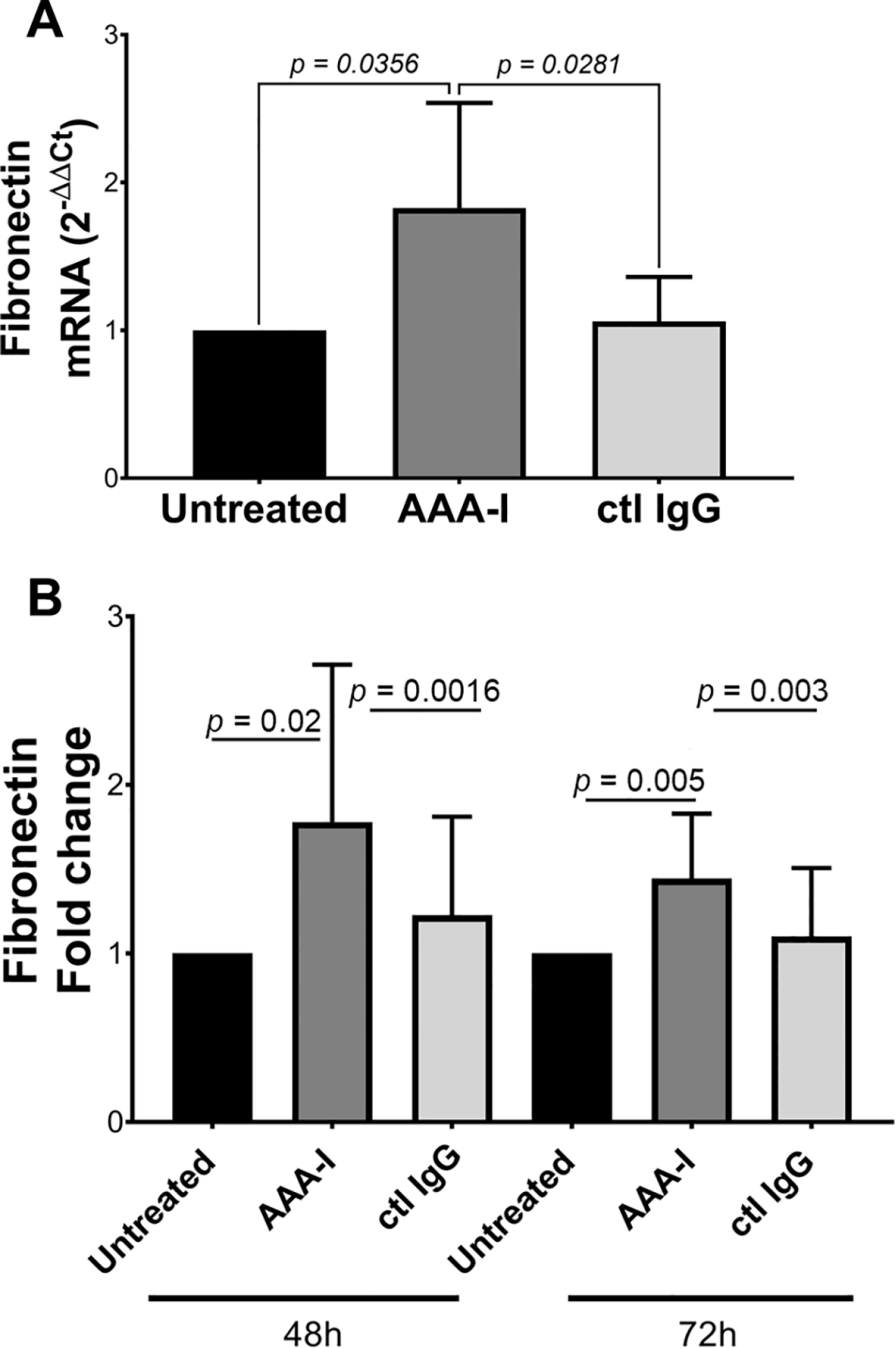
Evaluating the impact of AAA-I on LX-2 cells. **(A)** Fibronectin mRNA quantification in cells cultured in the presence/absence of AAA-I. **(B)** After 48 and 72 h, LX-2 cells cultured in the presence of AAA-I showed significantly increased concentrations of fibronectin compared to LX-2 cells alone and the LX-2/IgG control.

We sought to determine the effect of AAA-I on human hepatic stellate cells (HSC; LX-2) by measuring the concentration of fibronectin (FN), which is a crucial extracellular matrix protein that accumulates during liver fibrosis. We determined FN secreted into the cell culture supernatants and quantitated FN-mRNA in LX-2 cells cultured in the presence of AAA-I (**Figure 7**). LX-2 cells in the presence of AAA-I showed an 80% increase in FN-mRNA compared to LX-2 cells alone (*p* = 0.035) and the LX-2/IgG control (*p* = 0.028). After 48 h, LX-2 cells in the presence of AAA-I showed significantly increased levels of FN compared to LX2 cells alone (*p* = 0.02) and the LX-2/IgG control (*p* = 0.0016).

## Discussion

In this study, we have shown for the first time that individuals with spontaneous resolution of HCV infection, which occurs in a minority of those infected, have a similar AAA-I prevalence to that previously reported in a large population-based study (15)—i.e., 16% vs. 19.9% (*n* = 6,649). It has been shown that spontaneous clearance of HCV is associated with single nucleotide polymorphisms in MHC Class II genes (18). Individuals who clear acute HCV infection may not develop new-onset AAA-I, not only because of their immunogenetic background but also their short-lived HCV infection. In contrast, we have confirmed that individuals who progress to chronic HCV infection have a significantly higher prevalence of AAA-I (47%) and have shown that the magnitude of the response is higher in more advanced disease. We have also shown for the first time that AAA-I persists in individuals who have been cured with interferon-based antiviral therapy.

Our results support the hypothesis that CHC infection can induce the development of AAA-I. HCV viral entry, replication, assembly, and egress are tightly connected with host lipoproteins and apolipoproteins [reviewed in (19)]. ApoA-I is a component not only of highly infectious HCV lipoviral particles (LVP) (20) but also of “empty” LVP, which contain no HCV RNA but are HCV-modified lipoproteins within the triglyceride-rich lipoprotein (TRL) family (21). These HCV envelope glycoprotein-containing subviral particles are the predominant TRL in CHC. ApoA-I is the structural apolipoprotein of HDL, but proteomic analysis has also found apoA-I in VLDL and LDL (22), so apoA-I would be associated with both infectious LVP and subviral particles. Downregulation of apoA-I has also been shown to reduce both HCV RNA and viral particle levels *in vitro* (23), implying that apoA-I is required for the production of infectious LVP. ApoA-I is the principal component of HDL, and the amphipathic α-helices of apoA-I are critical for interaction with scavenger receptor class B type I (SR-BI), the endogenous liver receptor for HDL. SR-BI is also an entry factor for HCV (24). Amphipathic α-helix motifs in the exchangeable apolipoproteins have been shown to be a key structural factor in conferring viral infectivity (25).

**Figure 8:**
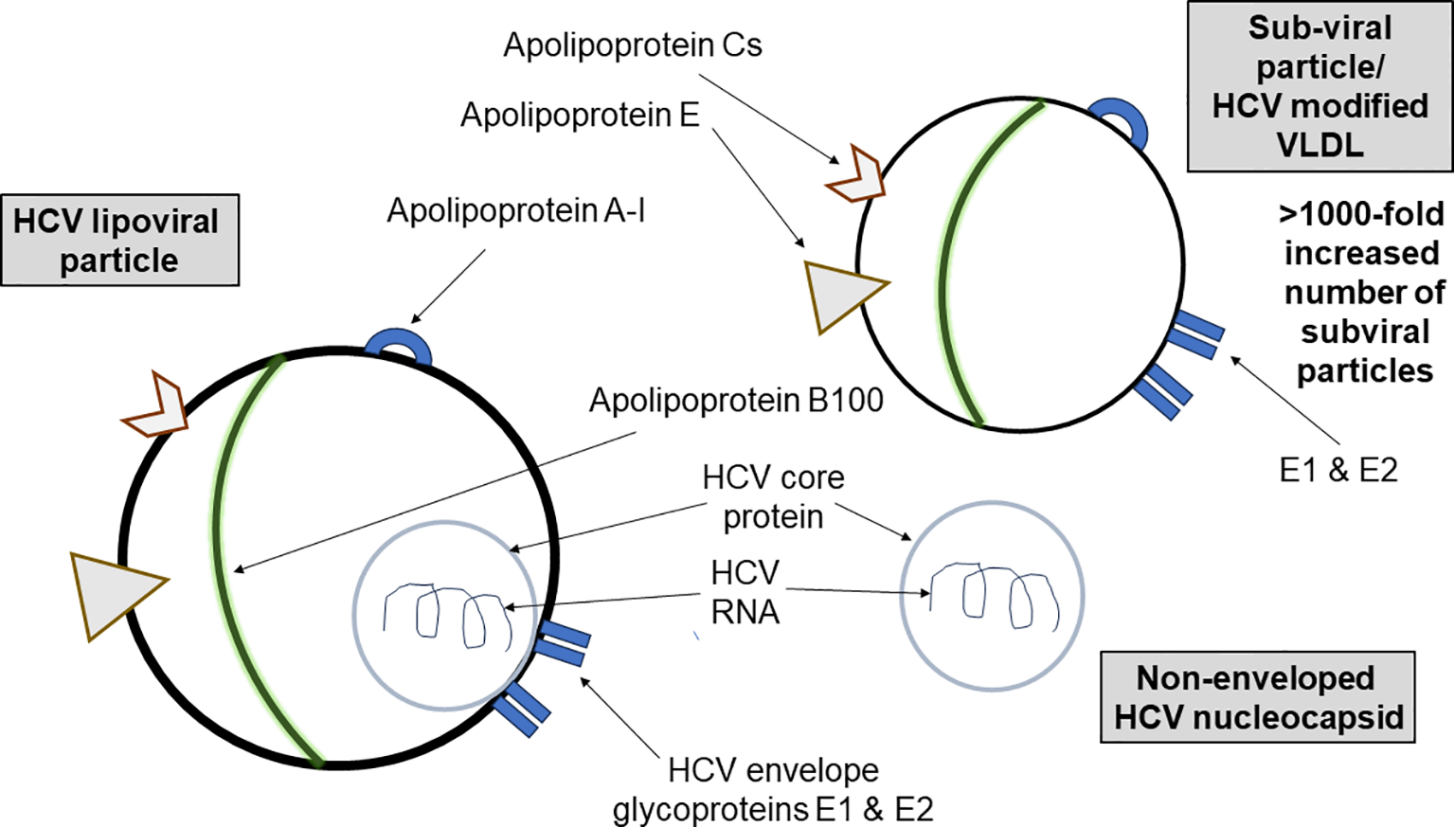
A schematic showing the diversity of HCV lipoviral particles and subviral particles found in the blood of patients with chronic HCV infection. HCV particles in the blood of infected patients are heterogeneous in size, density, and infectivity. There are more than a thousand-fold more nucleocapsid-free subviral particles containing apolipoprotein A-I than infectious apoA-I-containing lipoviral particles. Exposure to these viral and subviral particles may lead to autoreactivity.

These heterogeneous HCV particles (**Figure 8**) may lead to autoreactivity to host apoA-I through mechanisms such as epitope spreading and molecular mimicry [reviewed in (26)]. However, during viral infections, B cells recognizing virion epitopes endocytose and process virions for antigen presentation to CD4^+^ helper T (Th) cells specific for virion proteins (27). Consequently, the epitope-specific Th cells provide intermolecular help to numerous B-cell specificities (28). It is known that B-cell homeostasis and tolerance are disrupted in CHC patients, with an expansion of IgM memory B cells producing autoreactive antibodies and a decrease in the naïve B-cell population (29). Furthermore, this autoimmunity may be linked to increased numbers of regulatory T cells (T_reg_) and immunoregulatory cytokines (e.g., IL-10 and TGF-β), which contribute to impaired T-cell responses in CHC (30). It is of interest that HCV-specific T_reg_ cells persist after virological cure (31) and, as T_reg_ cells are pivotal in preventing autoimmunity, they may persist to control AAA-I and other HCV-induced autoimmune phenomena (2).

In this cohort of CHC patients, we found that AAA-I positivity and median AAA-I levels were higher in patients with cirrhosis. Furthermore, the presence of AAA-I was equivalent to HDL-related biomarkers (HDL-C and apoA-I) in predicting cirrhosis. Lower apoA-I is known to correlate with more fibrosis (32) and reduced apoA-I has also been shown to be a robust predictor of cirrhosis complications and survival (33). The finding that AAA-I is a similar biomarker implies a pathological role for this autoantibody. In a preliminary *in vitro* study, we found that AAA-I upregulated the synthesis and secretion of the extracellular matrix protein fibronectin in human HSCs, the predominant cell type responsible for liver fibrogenesis (34). AAA-I may also render apoA-I/HDL dysfunctional and impair their anti-inflammatory role (35). Conversely, AAA-I has been shown to be proinflammatory, possibly through innate immune receptor signaling via Toll-like receptors (TLR) 2- and 4-mediated pathways (36, 37), leading to increased interferon-α production by human-derived macrophages *in vitro* (8). It is noteworthy that chronic innate immune activation is the strongest negative predictor of response to interferon-α-based antiviral therapy [reviewed in (30)], the previous standard of care.

As AAA-I is proinflammatory, the presence of this autoantibody in some subjects after successful antiviral therapy may possibly account for the ongoing histological changes in the liver that have been reported in HCV antibody-positive, HCV RNA-negative individuals (38). Although Hoare et al. suggested that the presence of inflammatory infiltrate and fibrosis reflects persistent virus infection in the liver, the accompanying editorial questioned whether the histological changes could represent an “autoimmune-like” response (39). Furthermore, if the maintenance of an autoantibody response after successful antiviral therapy contributes to increased numbers of regulatory B and T cells (40), these may, in turn, impair cancer immunosurveillance and predispose individuals to the development of hepatocellular carcinoma.

We conclude that AAA-I is found in a substantial proportion of CHC patients. These autoantibodies are more frequent in advanced disease and can persist after virological cure. Individuals who clear acute HCV infection have a significantly lower frequency of AAA-I. These autoantibodies, previously shown to be a biomarker of CV risk and a predictor of all-cause mortality (6), also predict cirrhosis in CHC patients. Further research is warranted to explore the natural history of their evolution in individuals with CHC sequentially and after virological cure. Such studies could determine whether AAA-I positivity is associated with nondrug-related mortality among patients successfully treated for hepatitis C (5).

## Data Availability

The raw data supporting the conclusions of this article will be made available by the authors, without undue reservation.

## References

[1] Sharma C Bayry J. High risk of autoimmune diseases after COVID-19. Nat Rev Rheumatol. (2023) 19:399–400. doi: 10.1038/s41584-023-00964-y 37046064 PMC10096101

[2] Manns MP Rambusch EG. Autoimmunity and extrahepatic manifestations in hepatitis C virus infection. J Hepatol. (1999) 31 Suppl 1:39–42. doi: 10.1016/s0168-8278(99)80372-9 10622558

[3] Bartenschlager R Baumert TF Bukh J Houghton M Lemon SM Lindenbach BD. Critical challenges and emerging opportunities in hepatitis C virus research in an era of potent antiviral therapy: Considerations for scientists and funding agencies. Virus Res. (2018) 248:53–62. doi: 10.1016/j.virusres.2018.02.016 29477639

[4] Dhiman RK Premkumar M. Hepatitis C virus elimination by 2030: conquering mount improbable. Clin Liver Dis (Hoboken). (2020) 16:254–61. doi: 10.1002/cld.978 PMC7805299 33489098

[5] Hamill V Wong S Benselin J Krajden M Hayes PC Mutimer D. Mortality rates among patients successfully treated for hepatitis C in the era of interferon-free antivirals: population based cohort study. BMJ. (2023) 382:e074001. doi: 10.1136/bmj-2022-074001 37532284 PMC10394680

[6] Antiochos P Marques-Vidal P Virzi J Pagano S Satta N Hartley O. Anti-apolipoprotein A-1 IgG predict all-cause mortality and are associated with Fc receptor-like 3 polymorphisms. Front Immunol. (2017) 8:437. doi: 10.3389/fimmu.2017.00437 28458671 PMC5394854

[7] Bridge SH Pagano S Jones M Foster GR Neely D Vuilleumier N. Autoantibody to apolipoprotein A-1 in hepatitis C virus infection: a role in atherosclerosis? Hepatol Int. (2018) 12:17–25. doi: 10.1007/s12072-018-9842-5 29423541 PMC5814532

[8] L’Huillier AG Pagano S Baggio S Meyer B Andrey DO Nehme M. Autoantibodies against apolipoprotein A-1 after COVID-19 predict symptoms persistence. Eur J Clin Invest. (2022) 52:e13818. doi: 10.1111/eci.13818 35598178 PMC9348059

[9] Frias MA Virzi J Batuca J Pagano S Satta N Delgado Alves J. ELISA methods comparison for the detection of auto-antibodies against apolipoprotein A1. J Immunol Methods. (2019) 469:33–41. doi: 10.1016/j.jim.2019.03.011 30926534

[10] Preiss D Neely D. Biochemistry laboratories should routinely report non-HDL-cholesterol. Ann Clin Biochem. (2015) 52:629–31. doi: 10.1177/0004563215594818 26085052

[11] Pagano S Gaertner H Cerini F Mannic T Satta N Teixeira PC. The human autoantibody response to apolipoprotein A-I is focused on the C-terminal helix: A new rationale for diagnosis and treatment of cardiovascular disease? PLoS One. (2015) 10:e0132780. doi: 10.1371/journal.pone.0132780 26177543 PMC4503694

[12] Vuilleumier N Rossier MF Pagano S Python M Charbonney E Nkoulou R. Anti-apolipoprotein A-1 IgG as an independent cardiovascular prognostic marker affecting basal heart rate in myocardial infarction. Eur Heart J. (2010) 31:815–23. doi: 10.1093/eurheartj/ehq055 20176799

[13] Livak KJ Schmittgen TD. Analysis of relative gene expression data using real-time quantitative PCR and the 2(-Delta Delta C(T)) Method. Methods. (2001) 25:402–8. doi: 10.1006/meth.2001.1262 11846609

[14] Lloyd-Jones DM. Cardiovascular risk prediction: basic concepts, current status, and future directions. Circulation. (2010) 121:1768–77. doi: 10.1161/CIRCULATIONAHA.109.849166 20404268

[15] Antiochos P Marques-Vidal P Virzi J Pagano S Satta N Bastardot F. Association between anti-apolipoprotein A-1 antibodies and cardiovascular disease in the general population. Results from the CoLaus study. Thromb Haemost. (2016) 116:764–71. doi: 10.1160/TH16-03-0248 27384400

[16] Ziol M Handra-Luca A Kettaneh A Christidis C Mal F Kazemi F. Noninvasive assessment of liver fibrosis by measurement of stiffness in patients with chronic hepatitis C. Hepatology. (2005) 41:48–54. doi: 10.1002/hep.20506 15690481

[17] Degos F Perez P Roche B Mahmoudi A Asselineau J Voitot H. Diagnostic accuracy of FibroScan and comparison to liver fibrosis biomarkers in chronic viral hepatitis: a multicenter prospective study (the FIBROSTIC study). J Hepatol. (2010) 53:1013–21. doi: 10.1016/j.jhep.2010.05.035 20850886

[18] Valencia A Vergara C Thio CL Vince N Douillard V Grifoni A. Trans-ancestral fine-mapping of MHC reveals key amino acids associated with spontaneous clearance of hepatitis C in HLA-DQbeta1. Am J Hum Genet. (2022) 109:299–310. doi: 10.1016/j.ajhg.2022.01.001 35090584 PMC8874224

[19] Vieyres G Pietschmann T. HCV Pit Stop at the Lipid Droplet: Refuel Lipids and Put on a Lipoprotein Coat before Exit. Cells. (2019) 8(3):233. doi: 10.3390/cells8030233 30871009 PMC6468556

[20] Catanese MT Uryu K Kopp M Edwards TJ Andrus L Rice WJ. Ultrastructural analysis of hepatitis C virus particles. Proc Natl Acad Sci U.S.A. (2013) 110:9505–10. doi: 10.1073/pnas.1307527110 PMC3677472 23690609

[21] Scholtes C Ramiere C Rainteau D Perrin-Cocon L Wolf C Humbert L. High plasma level of nucleocapsid-free envelope glycoprotein-positive lipoproteins in hepatitis C patients. Hepatology. (2012) 56:39–48. doi: 10.1002/hep.25628 22290760

[22] Sun HY Chen SF Lai MD Chang TT Chen TL Li PY. Comparative proteomic profiling of plasma very-low-density and low-density lipoproteins. Clin Chim Acta. (2010) 411:336–44. doi: 10.1016/j.cca.2009.11.023 19945452

[23] Mancone C Steindler C Santangelo L Simonte G Vlassi C Longo MA. Hepatitis C virus production requires apolipoprotein A-I and affects its association with nascent low-density lipoproteins. Gut. (2011) 60:378–86. doi: 10.1136/gut.2010.211292 20940285

[24] Bartosch B Vitelli A Granier C Goujon C Dubuisson J Pascale S. Cell entry of hepatitis C virus requires a set of co-receptors that include the CD81 tetraspanin and the SR-B1 scavenger receptor. J Biol Chem. (2003) 278:41624–30. doi: 10.1074/jbc.M305289200 12913001

[25] Fukuhara T Wada M Nakamura S Ono C Shiokawa M Yamamoto S. Amphipathic alpha-helices in apolipoproteins are crucial to the formation of infectious hepatitis C virus particles. PloS Pathog. (2014) 10:e1004534. doi: 10.1371/journal.ppat.1004534 25502789 PMC4263759

[26] Sundaresan B Shirafkan F Ripperger K Rattay K. The role of viral infections in the onset of autoimmune diseases. Viruses. (2023) 15(3):782. doi: 10.3390/v15030782 36992490 PMC10051805

[27] Zinkernagel RM Pircher HP Ohashi P Oehen S Odermatt B Mak T. T and B cell tolerance and responses to viral antigens in transgenic mice: implications for the pathogenesis of autoimmune versus immunopathological disease. Immunol Rev. (1991) 122:133–71. doi: 10.1111/j.1600-065X.1991.tb00601.x 1937540

[28] Milich DR McLachlan A Thornton GB Hughes JL. Antibody production to the nucleocapsid and envelope of the hepatitis B virus primed by a single synthetic T cell site. Nature. (1987) 329:547–9. doi: 10.1038/329547a0 2443856

[29] Roughan JE Reardon KM Cogburn KE Quendler H Pockros PJ Law M. Chronic hepatitis C virus infection breaks tolerance and drives polyclonal expansion of autoreactive B cells. Clin Vaccine Immunol. (2012) 19:1027–37. doi: 10.1128/CVI.00194-12 PMC3393369 22623650

[30] Park SH Rehermann B. Immune responses to HCV and other hepatitis viruses. Immunity. (2014) 40:13–24. doi: 10.1016/j.immuni.2013.12.010 24439265 PMC4480226

[31] Langhans B Nischalke HD Kramer B Hausen A Dold L van Heteren P. Increased peripheral CD4(+) regulatory T cells persist after successful direct-acting antiviral treatment of chronic hepatitis C. J Hepatol. (2017) 66:888–96. doi: 10.1016/j.jhep.2016.12.019 28040549

[32] Imbert-Bismut F Ratziu V Pieroni L Charlotte F Benhamou Y Poynard T. Biochemical markers of liver fibrosis in patients with hepatitis C virus infection: a prospective study. Lancet. (2001) 357:1069–75. doi: 10.1016/S0140-6736(00)04258-6 11297957

[33] Trieb M Rainer F Stadlbauer V Douschan P Horvath A Binder L. HDL-related biomarkers are robust predictors of survival in patients with chronic liver failure. J Hepatol. (2020) 73:113–20. doi: 10.1016/j.jhep.2020.01.026 32061870

[34] Khomich O Ivanov AV Bartosch B. Metabolic hallmarks of hepatic stellate cells in liver fibrosis. Cells. (2019) 9(1):24. doi: 10.3390/cells9010024 31861818 PMC7016711

[35] Rye KA Barter PJ. Antiinflammatory actions of HDL: a new insight. Arterioscler Thromb Vasc Biol. (2008) 28:1890–1. doi: 10.1161/ATVBAHA.108.173575 18946054

[36] Montecucco F Braunersreuther V Burger F Lenglet S Pelli G Carbone F. Anti-apoA-1 auto-antibodies increase mouse atherosclerotic plaque vulnerability, myocardial necrosis and mortality triggering TLR2 and TLR4. Thromb Haemost. (2015) 114:410–22. doi: 10.1160/TH14-12-1039 25879306

[37] Pagano S Satta N Werling D Offord V de Moerloose P Charbonney E. Anti-apolipoprotein A-1 IgG in patients with myocardial infarction promotes inflammation through TLR2/CD14 complex. J Intern Med. (2012) 272:344–57. doi: 10.1111/j.1365-2796.2012.02530.x 22329401

[38] Hoare M Gelson WT Rushbrook SM Curran MD Woodall T Coleman N. Histological changes in HCV antibody-positive, HCV RNA-negative subjects suggest persistent virus infection. Hepatology. (2008) 48:1737–45. doi: 10.1002/hep.22484 PMC2680218 18925639

[39] Wiesner RH. Is there disease progression in patients who are hepatitis C virus antibody-positive and hepatitis C virus RNA-seronegative? Hepatology. (2008) 48:1734–6. doi: 10.1002/hep.22728 19026008

[40] Hetta HF Mekky MA Zahran AM Abdel-Malek MO Ramadan HK Shafik EA. Regulatory B cells and their cytokine profile in HCV-related hepatocellular carcinoma: association with regulatory T cells and disease progression. Vaccines (Basel). (2020) 8(3):380. doi: 10.3390/vaccines8030380 32664587 PMC7565874

