# Supplementary material for: Autoantibodies to apolipoprotein A-I in hepatitis C virus infection: a role in disease progression?
Source: Front Immunol. 2025 Mar 20;16:1461041. doi: 10.3389/fimmu.2025.1461041 (PMC11965114; doi:10.3389/fimmu.2025.1461041)
Supplement: Supplementary file 1 [file SupplementaryFile1.docx]

**Autoantibodies to apolipoprotein A-I in hepatitis C virus infection: a role in disease progression?**

Simon H. Bridge, Sabrina Pagano, John K. Lodge_,_ Isaac T. Shawa,

Paula Marin-Crespo, Matthew E. Cramp, David A. Sheridan,

Simon D. Taylor-Robinson, Nicolas Vuilleumier,

R. Dermot G. Neely & Margaret F. Bassendine

Supplementary material contents

[Figure S1 2](#_Toc142056656)

[Figure S2 4](#_Toc142056657)

[Figure S3 5](#_Toc142056658)

[Figure S4 6](#_Toc142056659)

[Figure S5 7](#_Toc142056660)

[References 7](#_Toc142056661)


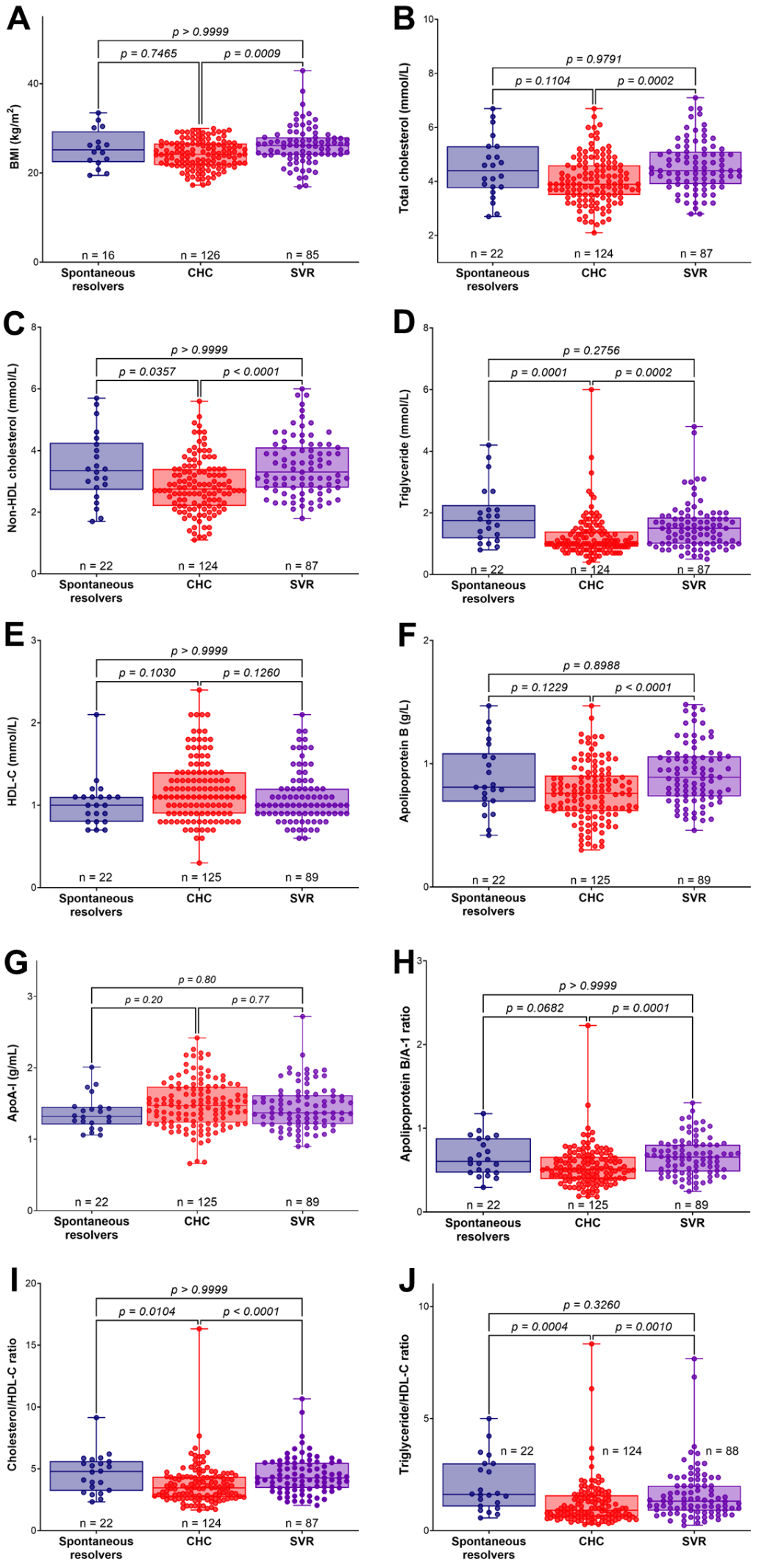


Figure S1. Multiple comparisons for continuous clinical and biochemical characteristics in spontaneous resolvers, chronic HCV infection and sustained virological responders. (A) Body mass index; Kruskal-Wallis ANOVA test with Dunn’s correction for multiple comparisons. (B) Total cholesterol; One-way ANOVA test with Sidak’s multiple comparison test. (C) Non-HDL-cholesterol; Kruskal-Wallis ANOVA test with Dunn’s correction for multiple comparisons. (D) Triglyceride; Kruskal-Wallis ANOVA test with Dunn’s correction for multiple comparisons. (E) HDL-C; Kruskal-Wallis ANOVA test with Dunn’s correction for multiple comparisons. (F) Apolipoprotein B; One-way ANOVA test with Sidak’s multiple comparison test. (G) Apolipoprotein A-I; Kruskal-Wallis ANOVA test with Dunn’s correction for multiple comparisons. (H) ApoB/ApoA-I ratio; Kruskal-Wallis ANOVA test with Dunn’s correction for multiple comparisons. (I) Cholesterol/HDL-C ratio; Kruskal-Wallis ANOVA test with Dunn’s correction for multiple comparisons and (J) Triglyceride/HDL-C ratio; Kruskal-Wallis ANOVA test with Dunn’s correction for multiple comparisons. All data points are shown and *p* values <0.05 were considered significant.

**
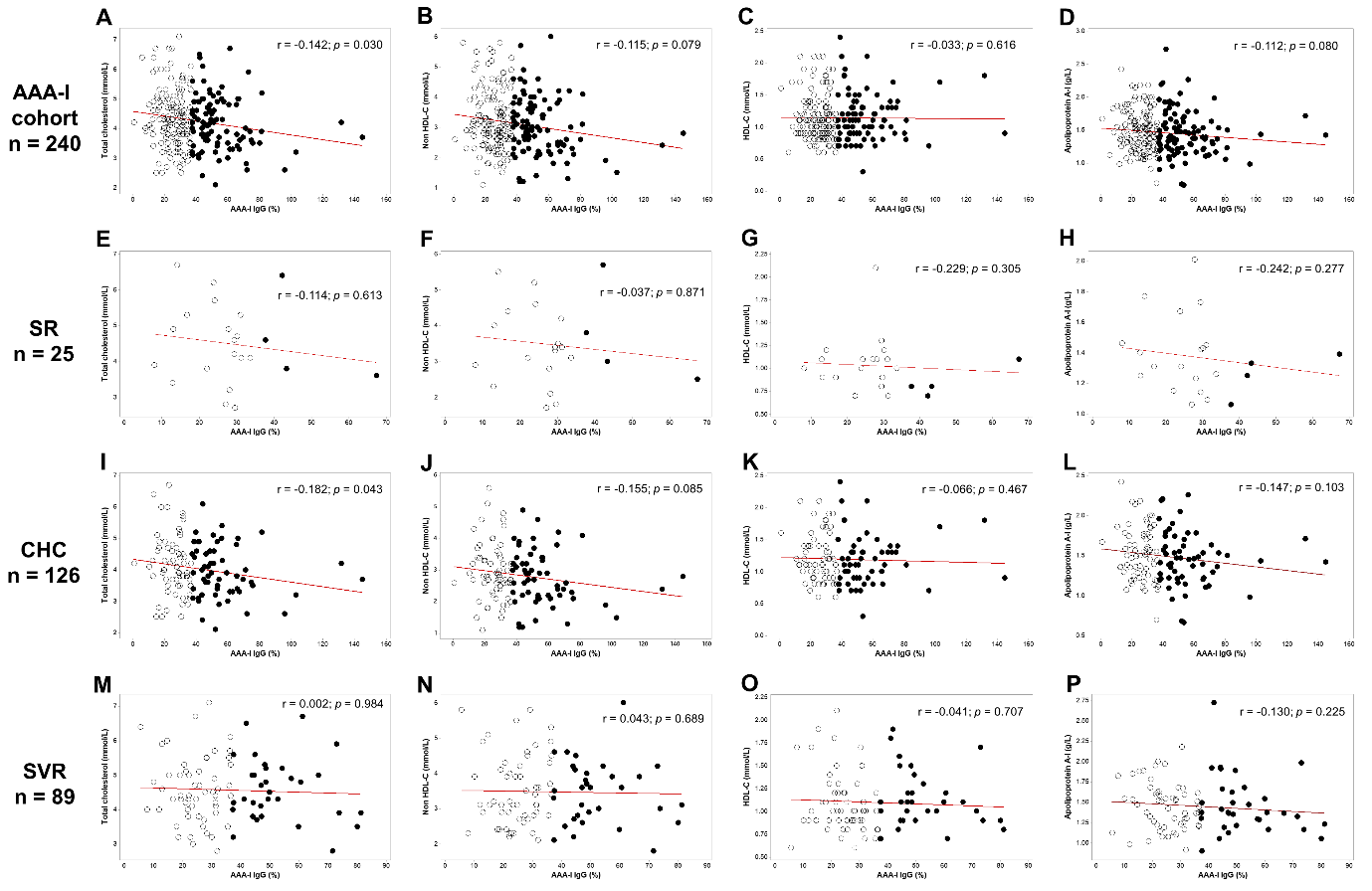
**

Figure S2. Scatterplots to show the association between AAA-I and lipoproteins (total cholesterol, non-HDL cholesterol and HDL-C) and apolipoprotein A-I. AAA-I cohort n = 240, SR cohort n = 25, CHC cohort n =126 and SVR cohort n = 89. (A) AAA-I cohort; total cholesterol *vs.* AAA-I. (B) AAA-I cohort; non-HDL-C *vs.* AAA-I. (C) AAA-I cohort; HDL-C *vs.* AAA-I. (D) AAA-I cohort; ApoA-I *vs.* AAA-I. (E) SR cohort; total cholesterol *vs.* AAA-I. (F) SR cohort; non-HDL-C *vs.* AAA-I. (G) SR cohort; HDL-C *vs.* AAA-I. (H) SR cohort; ApoA-I *vs.* AAA-I. (I) CHC cohort; total cholesterol *vs.* AAA-I. (J) CHC cohort; non-HDL-C *vs.* AAA-I. (K) CHC cohort; HDL-C *vs.* AAA-I. (L) CHC cohort; ApoA-I *vs.* AAA-I. (M) SVR cohort; total cholesterol *vs.* AAA-I. (N) SVR cohort; non-HDL-C *vs.* AAA-I. (O) SVR cohort; HDL-C *vs.* AAA-I and (P) SVR cohort; ApoA-I *vs.* AAA-I. The r and *p* values were calculated using a Spearman rank correlation test. Open circled samples were AAA-I IgG negative samples and closed black circles were AAA-I-positive samples. Broken red lines indicate the regression fit line.

**
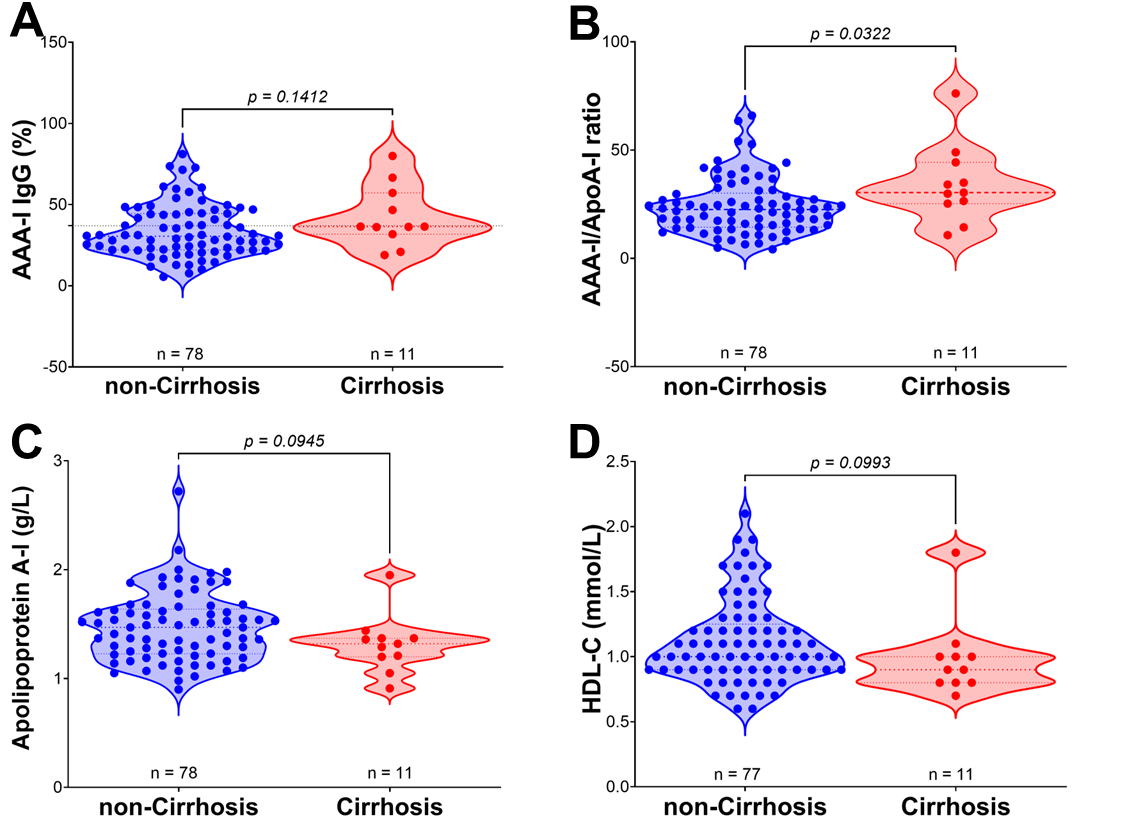
**

Figure S3. Comparison of AAA-I, AAA-I/ApoA-I ratio and HDL-related parameters in patients having achieved an SVR following antiviral treatment either without cirrhosis or with cirrhosis. (A) AAA-I IgG (%), 30.55% *vs.* 36.44%; Mann-Whitney test *p* = 0.1412 (B) AAA-I/ApoA-I ratio, 22.5 *vs.* 30.4; Mann-Whitney test *p* = 0.0322 (C) Apolipoprotein A-I, 1.47 g/L *vs.* 1.32 g/L; Welch’s t test *p* = 0.0945 and (D) HDL-C, 1.25 mmol/L *vs.* 1.00 mmol/L; Mann-Whitney test *p* = 0.0993.

**
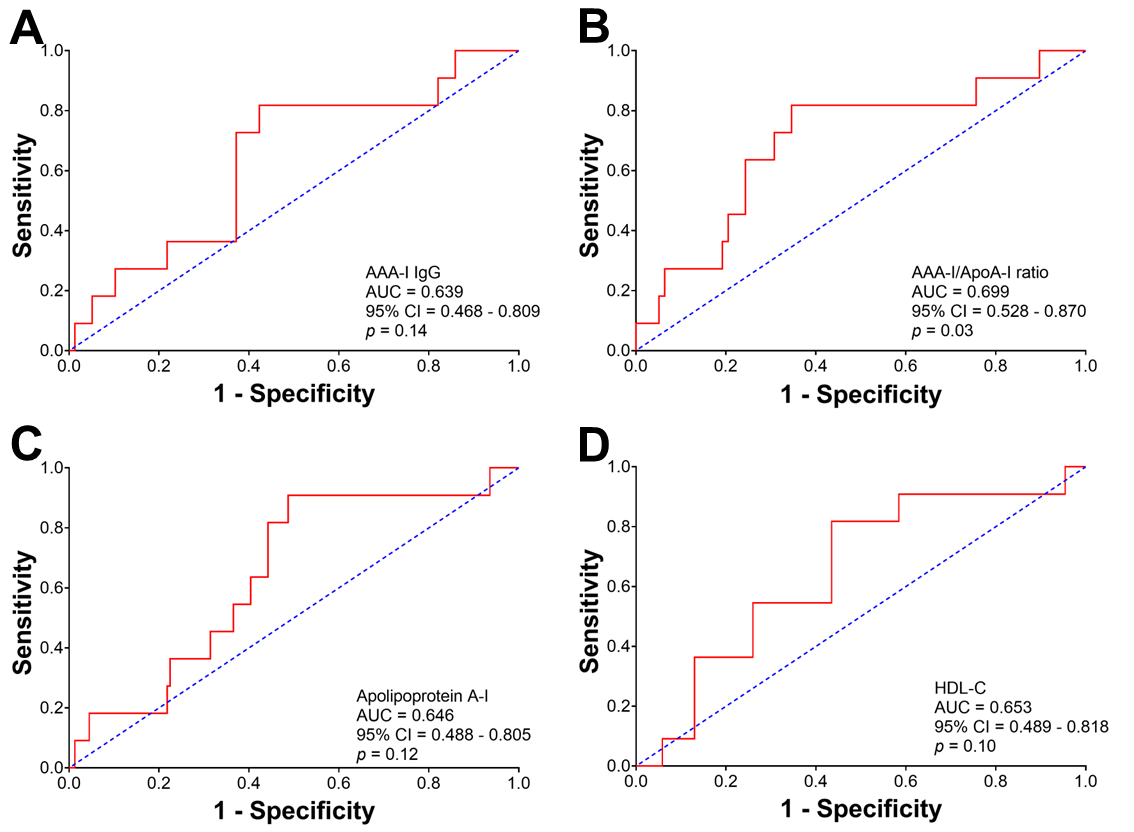
**

Figure S4. Receiver operating characteristic curve analysis to determine the robustness of AAA-I, AAA-I/ApoA-I ratio and HDL-related parameters at predicting cirrhosis in patients achieving an SVR after antiviral treatment. (A) AAA-I; AUC: 0.0.639, *p* = 0.14, (B) AAA-I/ApoA-I ratio; AUC: 0.699, *p* = 0.03, (C) Apolipoprotein A-I; AUC: 0.646, *p* = 0.12 and (D) HDL-C; AUC: 0.653, *p* = 0.10.

**
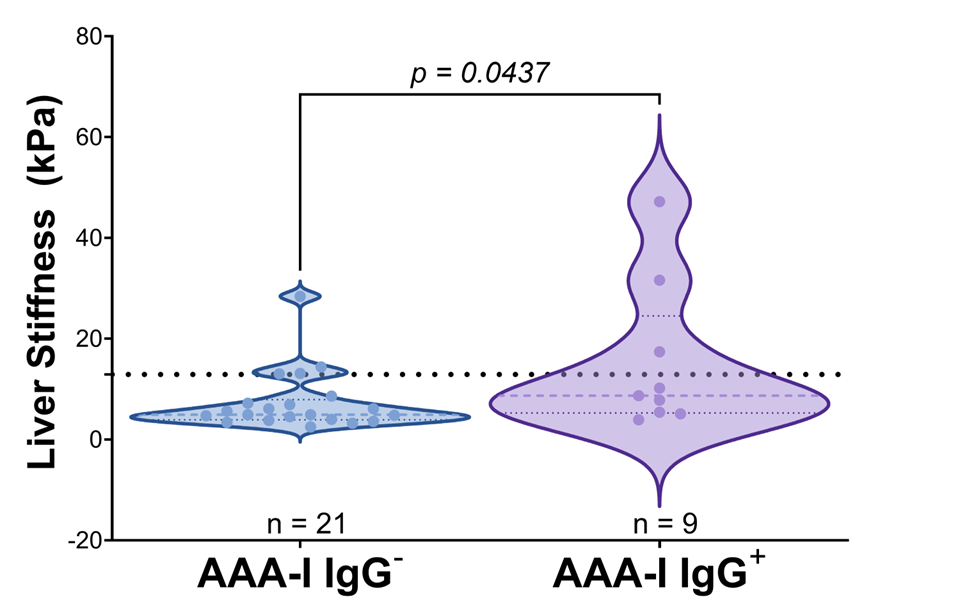
**

Figure S5. Violin plots to show the difference in liver stiffness measurements between AAA-I seronegative and seropositive individuals. The *p* value was calculated using the Mann-Whitney test. A *p* value <0.05 was the threshold of significance. The dotted line denotes the cut-off of >12.9 kPa for cirrhosis [1].

References

1. Degos F, Perez P, Roche B, Mahmoudi A, Asselineau J, Voitot H, et al. Diagnostic accuracy of FibroScan and comparison to liver fibrosis biomarkers in chronic viral hepatitis: a multicenter prospective study (the FIBROSTIC study). J Hepatol. 2010;53(6):1013-21. <https://doi.org/10.1016/j.jhep.2010.05.035> PMID: 20850886
